# Supplementary material for: Increased influenza severity in children in the wake of SARS‐CoV‐2
Source: Influenza Other Respir Viruses. 2023 Jul 23;17(7):e13178. doi: 10.1111/irv.13178 (PMC10363782; doi:10.1111/irv.13178)
Supplement: Supplementary file 1 — Table S1. Influenza Testing Criteria in NPICS Table S2. Descriptive and Clinical Characteristics of Participants Table S3. A/H3N2 symptom comparison, pre‐2020 vs. 2022 Table S4. A/H3N2 severe illness, by age group Table S5a. Odds ratios of influenza A/H3N2 severity, adjusting for potential confounders Table S5b. Odds ratios of influenza A/H3N2 severity in 0–4‐year‐olds, excluding those with comorbidities Table S6. B/Victoria symptom comparison, pre‐2020 vs. 2022 Table S7. B/Victoria severe illness, by age group Table S8. Odds ratios of influenza B/Victoria severity, adjusting for potential confounders Figure S1. Clinic visits of NPICS participants [file IRV-17-e13178-s001.docx]

Supplement for:

Increased Influenza Severity in Children in the Wake of SARS-CoV-2

Gregory Hoy^1^; Hannah E. Maier^1^; Guillermina Kuan^2,3^; Nery Sánchez^2^; Roger López^2,4^; Alyssa Meyers^1^; Miguel Plazaola^2^; Sergio Ojeda^2^; Angel Balmaseda^2,4^; Aubree Gordon^1^

^1^Department of Epidemiology, School of Public Health, University of Michigan, Ann Arbor, Michigan, USA; ^2^Sustainable Sciences Institute, Managua, Nicaragua; ^3^Centro de Salud Sócrates Flores Vivas, Ministry of Health, Managua, Nicaragua; ^4^Laboratorio Nacional de Virología, Centro Nacional de Diagnóstico y Referencia, Ministry of Health, Managua, Nicaragua

List of Figures and Tables:

Supplemental Table 1. Influenza Testing Criteria in NPICS

Supplemental Table 2. Descriptive and Clinical Characteristics of Participants

Supplemental Table 3. A/H3N2 symptom comparison, pre-2020 vs. 2022

Supplemental Table 4. A/H3N2 severe illness, by age group

Supplemental Table 5a. Odds ratios of influenza A/H3N2 severity, adjusting for potential confounders

Supplemental Table 5b. Odds ratios of influenza A/H3N2 severity in 0-4-year-olds, excluding those with comorbidities

Supplemental Table 6. B/Victoria symptom comparison, pre-2020 vs. 2022

Supplemental Table 7. B/Victoria severe illness, by age group

Supplemental Table 8. Odds ratios of influenza B/Victoria severity, adjusting for potential confounders

Supplemental Figure 1. Clinic visits of NPICS participants


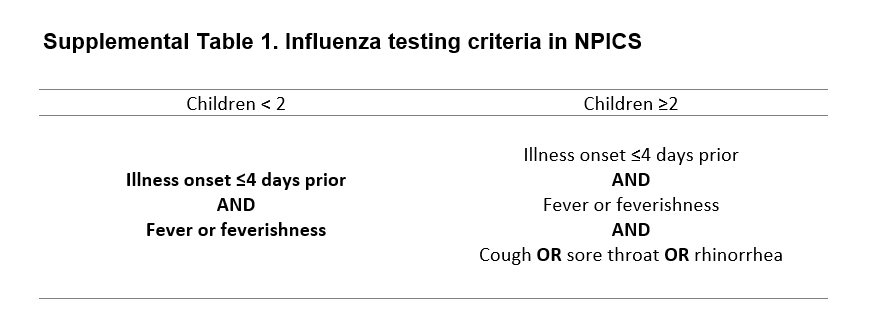


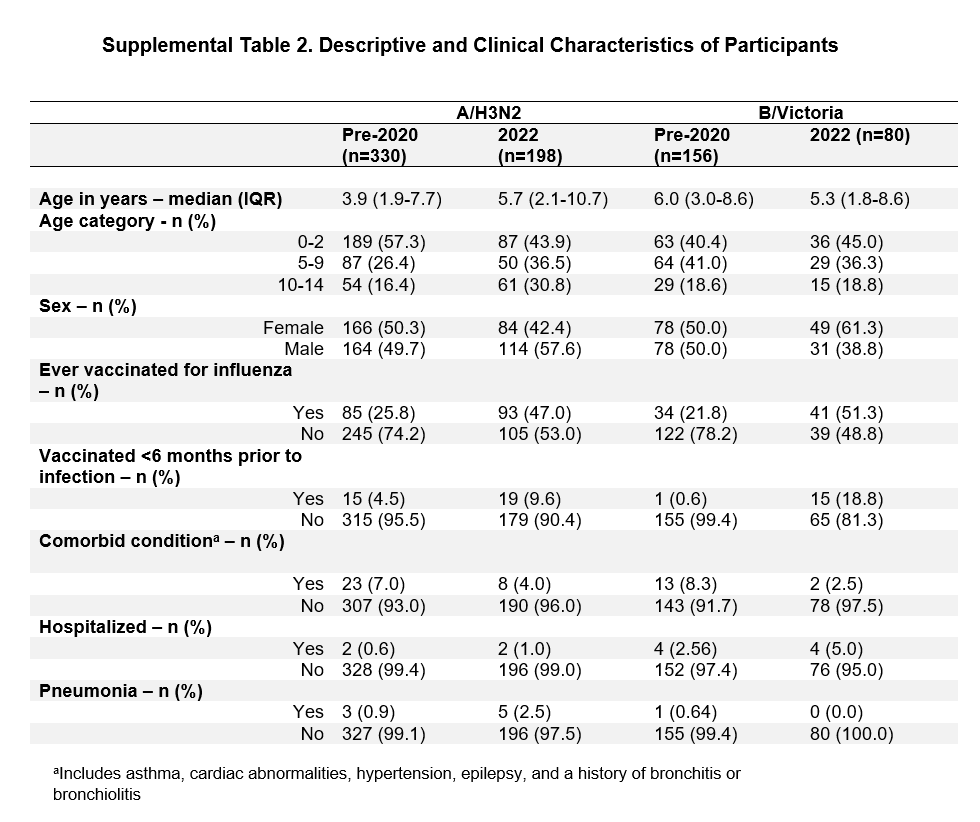


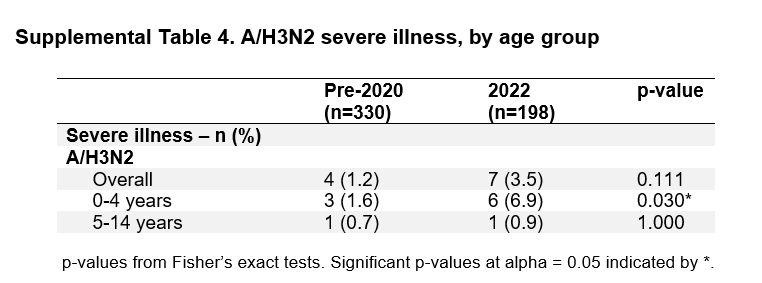

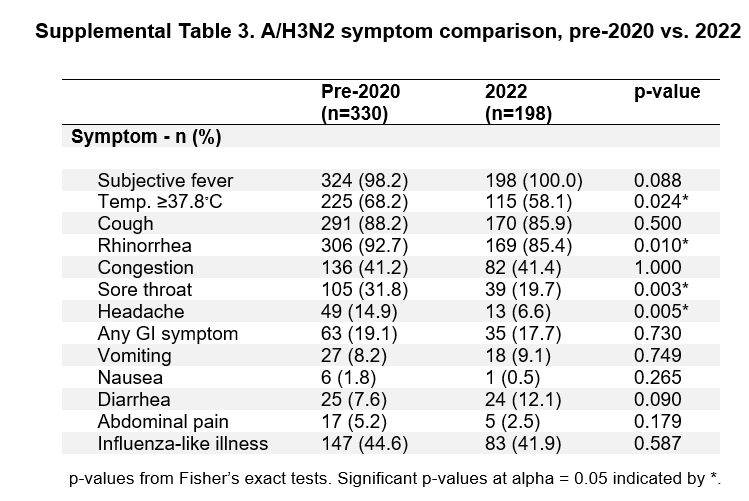


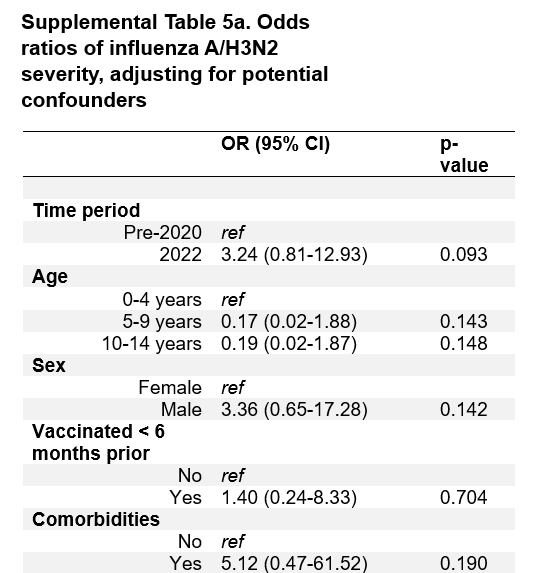


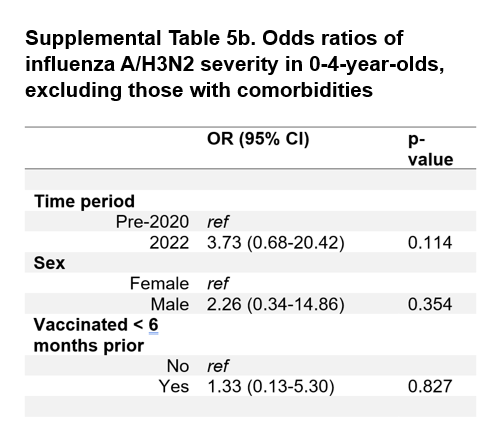


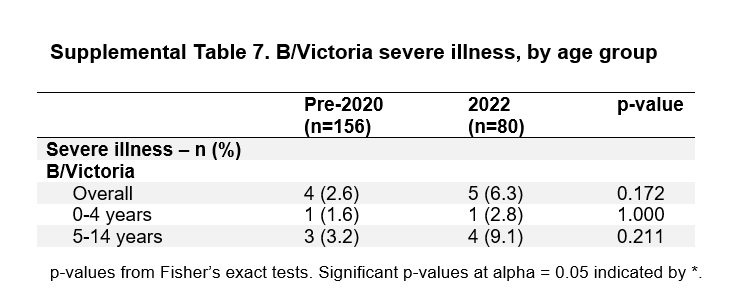

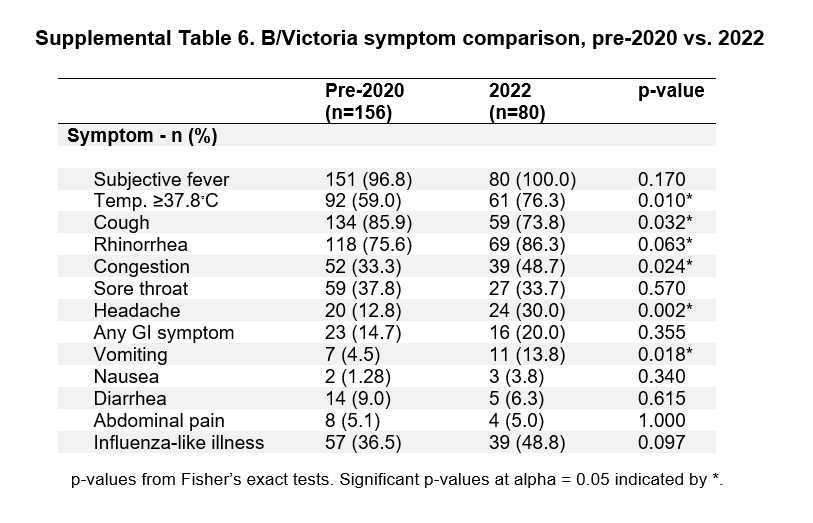


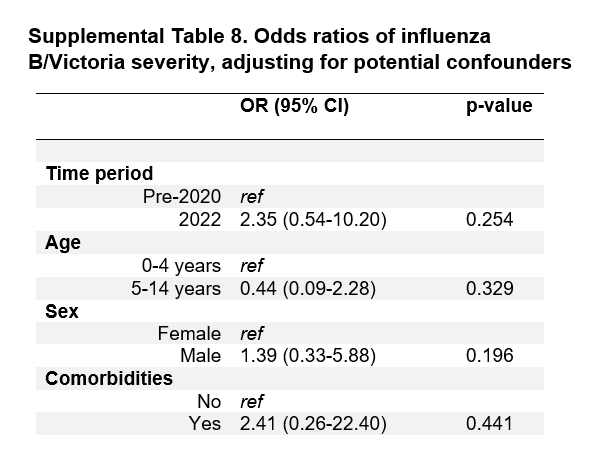


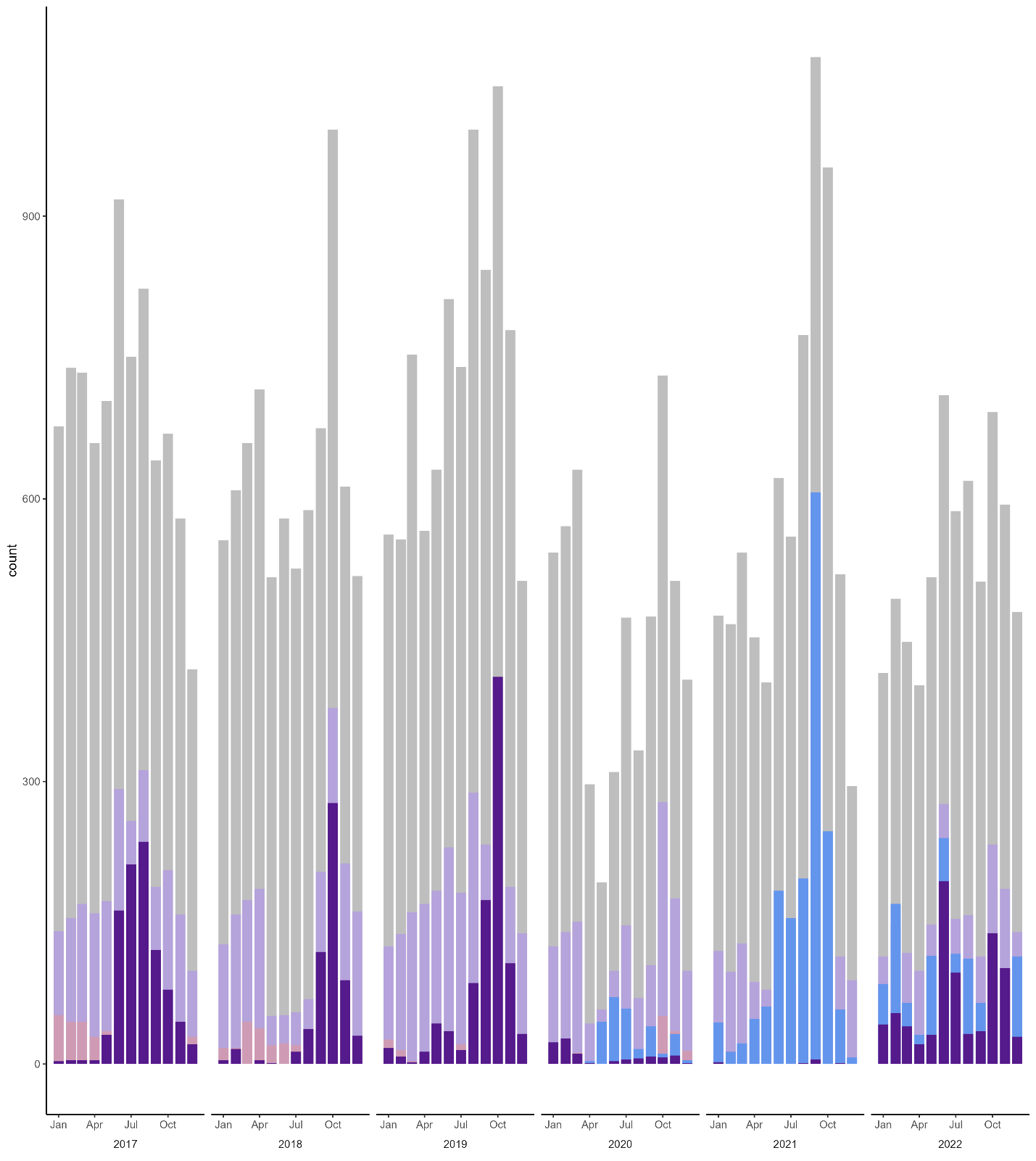
**Supplemental Figure 1. Clinic visits of NPICS participants**

Supplemental Figure 1. Clinic visits of NPICS participants. Total (respiratory and non-respiratory) visits (grey), visits that met influenza testing criteria (light purple), visits with PCR-confirmed influenza (dark purple), visits with PCR-confirmed SARS-CoV-2 (light blue), and visits with ALRI (light orange).
